# Artificial intelligence-driven assessment of salt caverns for underground hydrogen storage in Poland

**DOI:** 10.1038/s41598-024-64020-9

**Published:** 2024-06-20

**Authors:** Reza Derakhshani, Leszek Lankof, Amin GhasemiNejad, Mojtaba Zaresefat

**Affiliations:** 1 Department of Earth Sciences, Utrecht University, Utrecht, The Netherlands; 2 Department of Geology, Shahid Bahonar University of Kerman, Kerman, Iran; 3 Mineral and Energy Economy Research Institute of the Polish Academy of Sciences, Wybickiego 7A, 31-261 Krakow, Poland; 4 Department of Economics, Faculty of Management and Economics, Shahid Bahonar University of Kerman, Kerman, Iran; 5 Copernicus Institute of Sustainable Development, Utrecht University, Utrecht, The Netherlands

**Keywords:** Energy storage, Hydrogen storage

## Abstract

This study explores the feasibility of utilizing bedded salt deposits as sites for underground hydrogen storage. We introduce an innovative artificial intelligence framework that applies multi-criteria decision-making and spatial data analysis to identify the most suitable locations for storing hydrogen in salt caverns. Our approach integrates a unified platform with eight distinct machine-learning algorithms—KNN, SVM, LightGBM, XGBoost, MLP, CatBoost, GBR, and MLR—creating rock salt deposit suitability maps for hydrogen storage. The performance of these algorithms was evaluated using various metrics, including Mean Squared Error (MSE), Mean Absolute Error (MAE), Mean Absolute Percentage Error (MAPE), Root Mean Square Error (RMSE), and Correlation Coefficient (R^2^), compared against an actual dataset. The CatBoost model demonstrated exceptional performance, achieving an R^2^ of 0.88, MSE of 0.0816, MAE of 0.1994, RMSE of 0.2833, and MAPE of 0.0163. The novel methodology, leveraging advanced machine learning techniques, offers a unique perspective in assessing the potential of underground hydrogen storage. This approach is a valuable asset for various stakeholders, including government bodies, geological services, renewable energy facilities, and the chemical/petrochemical industry, aiding them in identifying optimal locations for hydrogen storage.

## Introduction

Hydrogen is touted as one of the foremost environmentally friendly fuels, emerging as a potent clean energy carrier^1–3^. With the advent and enhancement of renewable energy sources (RESs), the costs associated with electricity production are on a downward trajectory, ensuring medium-term benefits^4–6^. Notably, using surplus, low-cost renewable electricity for hydrogen conversion offers the dual advantage of storage for later use and significant economic efficiency augmentation of renewable energy systems^7^.

RESs, however, suffer from intermittency, highlighting an urgent need for robust hydrogen storage solutions^8,9^. Geological structures, encompassing rock salt deposits, depleted hydrocarbon deposits, and aquifers, emerge as viable candidates for large-scale hydrogen storage^10,11^. While preliminary assessments of such structures are available, they often overlook vital surface and underground factors, potentially limiting their suitability for storage^12^.

Recent years have witnessed a surge in the application of artificial intelligence algorithms, particularly machine learning, as formidable computational tools to simulate intricate phenomena across academic spectra^13–15^. The strength of these tools, especially artificial neural networks (ANN), lies in their innate learning capabilities, obviating the need for statistical source data assumptions and their proficiency in handling non-linear scenarios^16–18^. In recent years, machine learning methods have been increasingly used in research related to underground hydrogen storage in geological structures. The effective use of algorithms in predicting the values of critical parameters such as wettability affecting the storage capacity of porous rocks has been confirmed by numerous studies^19–21^. ML algorithms were also successfully used to predict interfacial tension in brine-hydrogen systems^22–26^. Research conducted by^27–30^ also focuses on developing data-driven ML models for predicting hydrogen solubility in water and brines under various pressure and temperature conditions. Research on the use of ML also concerns the optimization of hydrogen storage parameters and the design of energy systems supported by underground energy storage^31–33^, as well as the characterization of hydrogen storage reservoirs consisting of the prediction of thermodynamic parameters^34,35^. The convergence of machine learning and Geographic Information Systems (GIS) stands out as a game-changer, offering unprecedented insights into optimal locations for underground hydrogen storage (UHS).

Although UHS is a frequently discussed topic with numerous reviews available^36–42^, the meticulous assessment of storage potentials and associated technicalities remains paramount. Salt caverns, especially, emerged as the leading contenders for UHS, given their adoption in industrial contexts, such as the petrochemical industry^43–46^. Their storage efficacy is evidenced by operational examples like Teesside in the UK and locations in the USA like Clemens, Moss Bluff, and Spindletop^47^. Additionally, research endeavours worldwide emphasize rock salt deposits' immense hydrogen storage potential^48–56^. These caverns' sheer size and adaptable shape as well as rock salt's tightness and inert properties with respect to hydrogen, make them suitable for storing colossal hydrogen volumes^57–64^.

The technological intricacies involved in UHS in salt caverns are manifold, including evaluating cavern dimensions, rock salt properties, and the associated impact on storage capacity^58–64^. Beyond these technological considerations, UHS site selection is critically determined by rock salt deposit characteristics, like thickness and depth. A holistic approach for optimal site determination incorporates environmental, technical, economic, and social criteria^65,66^. The innovative integration of GIS with ML streamlines site selection and impact assessment, with successful applications observed in diverse fields^17,66–73^.

Our research introduces an innovative artificial intelligence framework, combining eight distinct machine-learning algorithms to generate suitability maps for rock salt deposit-based hydrogen storage. This study is a pioneering effort in the domain by harnessing the power of machine learning and complementing it with spatial data analysis. This methodology offers enhanced accuracy in determining hydrogen storage potential and equips stakeholders with an indispensable tool, potentially revolutionizing the decision-making process for hydrogen storage locations.

## Materials and methods

**Figure 1:**
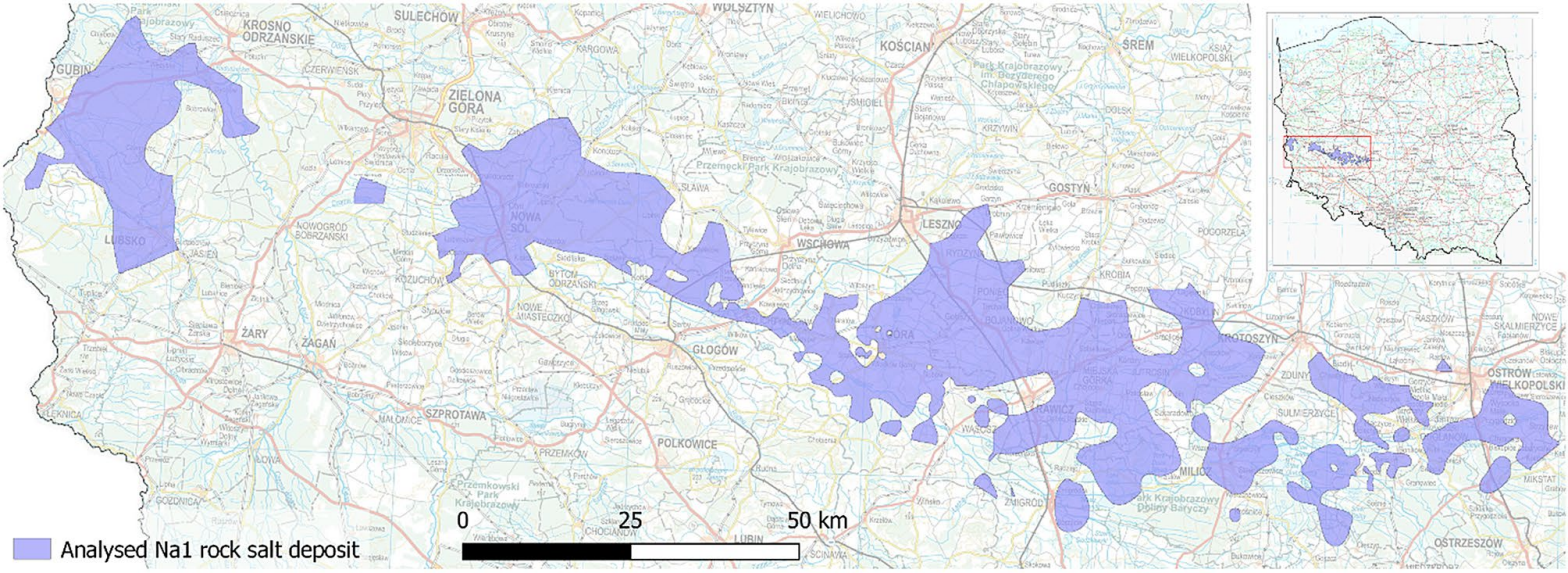
The Na1 rock salt deposit selected for analysis^76^ using ArcGIS Pro 2.8 software. The base map was developed by Esri using HERE data, DeLorme base map layers, OpenStreetMap contributors, Esri base map data, and select data from the GIS user community. For more information about Esri® software, please visit http://www.esri.com.

This study developed a comprehensive methodology to identify optimal locations for Underground Hydrogen Storage (UHS) within rock salt formations, focusing on the Na1 rock salt deposit in the Fore-Sudetic Monocline, southwest Poland. The Na1 unit, a part of the Upper Permian rock salt bearing formation extending across the Polish Lowland, was chosen for its favourable characteristics for hydrogen storage^53,74,75^. A part of the Na1 rock salt deposit with a thickness of over 130 m, occurring up to 1,800 m below ground level, was selected for analysis (Fig. 1).

### Methodological framework

- Overview of Integrated Approach

The methodology integrated Artificial Intelligence (AI) algorithms, Multi-Criteria Decision Analysis (MCDA), and Geographic Information System (GIS) spatial analysis. The Analytic Hierarchy Process (AHP) was employed to break down this complex issue into manageable components, establishing evaluation criteria, their weights, and a conclusive ranking of site alternatives.

The process entailed:

Defining Evaluation Criteria: Establishing the parameters for UHS site selection.

AI Algorithm Integration: Implementing eight machine-learning algorithms (KNN, SVM, LightGBM, XGBoost, MLP, CatBoost, GBR, and MLR) on a unified platform.

Data Segmentation: Dividing criteria-based data into a training set (70%) and a validation-testing set (30%).

Performance Assessment: Evaluating algorithmic performance using standard error metrics and the Correlation Coefficient (R^2^).

Optimal Algorithm Selection: Choosing the most effective algorithm based on performance metrics.

GIS Visualization: Mapping the spatial distribution of potential UHS sites.

Suitability Mapping: Creating a UHS suitability map from the selected algorithm's outputs.

Final Algorithm Formulation: Establishing a protocol for future research applications.

- Exclusion and Evaluation Criteria

The study incorporated both exclusion and evaluation criteria. Exclusion criteria, guided by Polish environmental regulations, eliminated areas within protected zones, residential and industrial areas, transportation networks, bodies of water, and active mining sites. Evaluation criteria focused on the rock salt layer's storage capacity, land development, access to water resources, road infrastructure, proximity to gas pipelines, energy demand, and the level of geological exploration.

### Data resources and preparation

- Data Collection

The study employed twelve standardized raster maps, with each map corresponding to a specific evaluative criterion. These criteria encompass hydrogen storage capacity, hydrological features, transportation infrastructure, gas pipeline network, land use development above the deposit, energy consumption across administrative units, and locations of geological research boreholes. The storage capacity map was developed by Lankof and Tarkowski^53^, while the remaining maps were acquired from various spatial information portals and the National Transmission System. All maps were harmonized in terms of extent and pixel specifications, ensuring consistency in pixel size and dimensions across the dataset.

- Map Transformation and Criteria Weighting

The hydrological features, transportation infrastructure, gas pipeline, and boreholes maps were transformed into proximity maps and then, together with other maps, normalized to a 1–10 scale, with higher values indicating greater suitability for UHS. The AHP method facilitated pairwise criteria comparison to establish weights, incorporating expert opinions from various fields.

### Machine learning algorithms overview

Artificial Intelligence (AI) is a branch of computer science dedicated to creating intelligent systems capable of learning and improving from experience. Machine learning (ML) is a critical domain within AI, exploring how systems can autonomously improve their performance. ML includes various techniques like representation learning and deep learning. These methods enable machines to automatically discover patterns in raw data and learn representations necessary for tasks such as detection or classification. The advancement of big data and AI technologies, especially in GPU computing power, has significantly impacted geological sciences. AI applications in geology include geological surveys, mineral recognition, and geochemical anomaly detection. This study focuses on using AI to evaluate potential sites for Underground Hydrogen Storage (UHS) in geological formations. Acknowledging the indispensable role of preprocessing in enhancing model reliability, we refined our dataset through systematic cleaning, normalization, and feature engineering processes. This ensured that our ML models were trained on data that accurately represented the underlying geological phenomena, laying a solid foundation for trustworthy estimations.

- *K-Nearest Neighbours (KNN)* The KNN is an intuitive and straightforward machine-learning algorithm for regression and classification^77^. It is an idle algorithm in machine learning as a new data mining method. In fact, it learns nothing from the training dataset rather than considering the features of the k-closest neighbors on the training dataset and calculating their distance. In other words, the KNN considers a point and all its nearby points in the training dataset. Thus, the distance from the point of forecasting in the testing dataset to the nearby points is calculated to define the closest neighbors. Ultimately, the same features and attributes are assigned to the forecasting point.
- *Support Vector Machine (SVM)* A robust algorithm for regression and classification tasks,^78,79^ SVM includes a unique parameter, ε, determining the width of the margin around the decision boundary, optimizing forecast accuracy^80–82^. The main goal of the Support Vector Machine (SVM) is to find the best possible dividing line, or 'hyperplane,' which creates the widest possible gap between distinct categories of data points. It is capable of interpreting both straightforward and complex patterns by employing specialized functions known as kernels. Widely utilized across various fields, particularly in the study of Earth sciences, this algorithm is renowned for its superior accuracy and reliability^83^.
- *Light Gradient Boosting Machine (LightGBM)* As another gradient-boosting framework, LightGBM concentrates on speed and efficiency. Therefore, a new tree-building algorithm is introduced known as gradient-based one-sided sampling (GOSS) for reducing the number of data during training. A histogram-based method is also used in LightGBM to bucket continuous properties within discrete bins. Thus, memory efficiency and training speed are enhanced while supporting distributed and parallel computing for large-scale datasets. The LightGBM is applied successfully in different domains, such as recommender systems, online advertising, and fraud detection.
- *Extreme Gradient Boosting (XGBoost)* XGBoost is a strong gradient-boosting framework with considerable performance and speed. Hence, a powerful predictive model can be made through the integration of weak learners, characteristically decision trees. To construct the XGBoost model, a stage-wise method is used, in which each following tree tries to correct the errors created by previous trees. XGBoost used gradient descent optimization methods during training for minimizing a precise loss function. The accuracy, scalability, and interpretability of XGBoost have critical roles in its extensive adoption across different domains, such as anomaly detection, click prediction, and web analytics.
- *Multilayer Perceptron (MLP)* Recently, a huge deal of attention has been attracted by neural networks^84^. ANNs are stimulated by biological neural networks to make non-linear models between dependent and independent variables, rivaling the learning of the biological neuron system^85^. An MLP is a kind of feed-forward neural network that includes multiple layers of interconnected artificial neurons. A non-linear activation function is applied by a neuron to the weighted sum of its inputs. Any arbitrary function can be approximated by MLPs considering adequate hidden units and proper activation functions^86^.
- *Categorical Boosting (CatBoost)* Various general implementation problems are addressed by the CatBoost technique for gradient boosting and solving the issue by offering ordering principles. Dorogush et al.^87^ developed CatBoost as an enhanced GBDT toolkit the same as XGBoost. The problems of gradient bias and prediction shift are solved by CatBoost. It has numerous advantages such as embedding an innovative algorithm to treat categorical features automatically as numerical characteristics. Moreover, it utilizes a combination of category properties, taking advantage of the connections between features and, importantly, enriching feature dimensions. Also, a perfectly symmetrical tree model is adopted to decrease overfitting and enhance the generalizability and accuracy of the algorithm.
- *Gradient Boosting Regressor (GBR)* GBR with better stability and higher performance is an integrated model. It was proposed by Friedman to extend the boosting algorithm and solve the regression problems. The negative gradients of the loss function are used to solve the minimum value in this algorithm. By Gradient Boosting, random differentiable loss functions are optimized thus constructing an additive model in a forward stage wise procedure. A regression tree in each stage fits the non-positive gradient of the presented loss function.
- *Multiple Linear Regression (MLR)* MLR models the linear relationship between multiple independent variables and a dependent variable, optimizing the fit through the minimization of a loss function.

### AI approach in UHS site evaluation

Our approach utilized the Fuzzy Analytic Hierarchy Process (FAHP) model to generate a target database for training the ML algorithms. In this paper, the input parameters of the ML algorithms included Conservation Area, Geological exploration, Water reservoir, Accessibility, Ecological Site, Energy Consumption, Land Use, Natural Gas Pipelines, Natural forest, Protected Area, Special Protection Area, Storage Capacity, and the AHP output derived from Lankof and Tarkowski^88^ as the ML algorithms output parameter.

A representative sample from the study area, comprising 1000 evenly spaced points, was selected for this purpose. The data was divided into a training set (70%) and a validation-testing set (30%).

**Figure 2:**
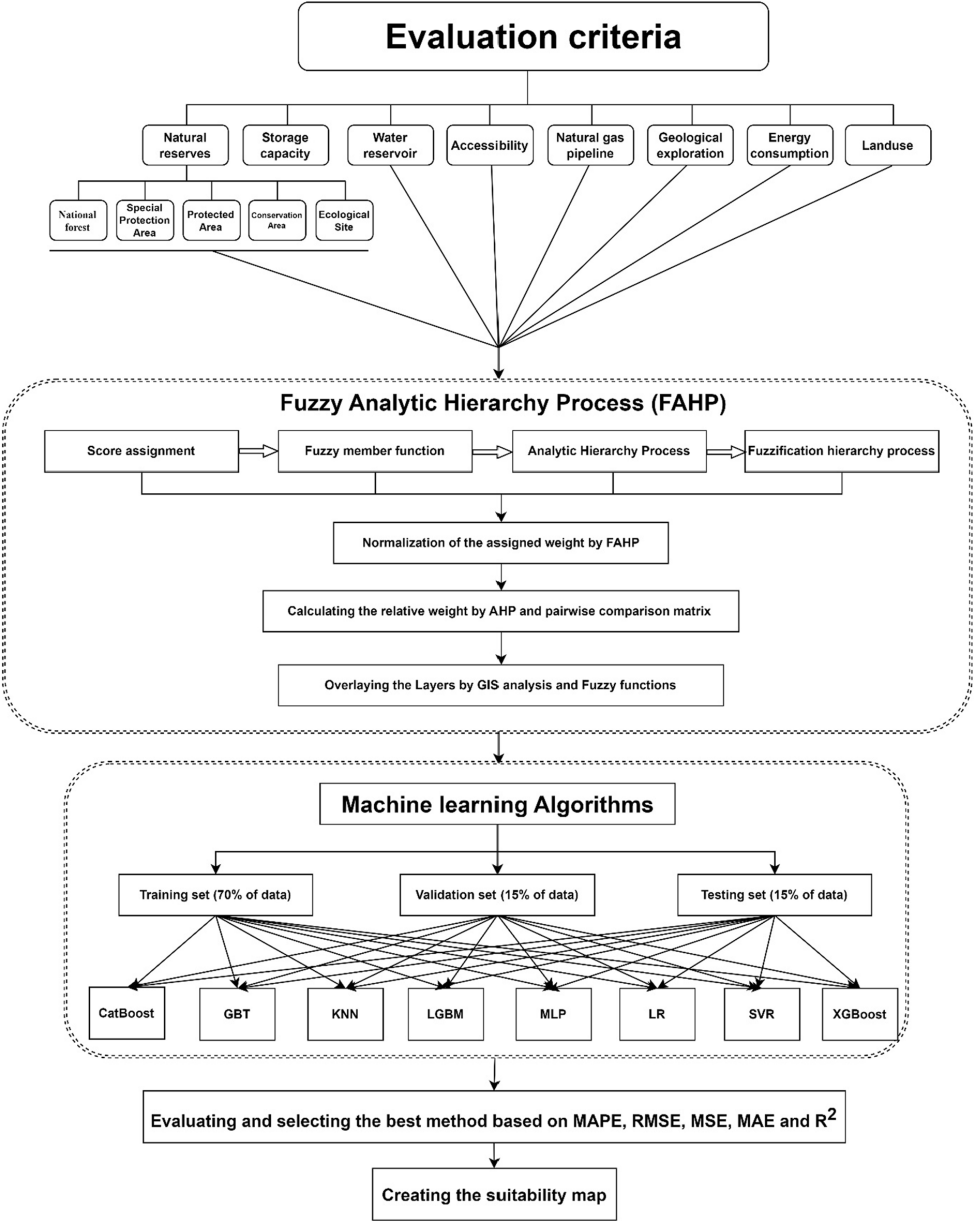
The workflow of the methodology.

The methodology employed in the present study is captured in Fig. 2, which outlines a multi-stage process integrating both Fuzzy Analytic Hierarchy Process (FAHP) and various Machine Learning (ML) algorithms to create a suitability map in rock salt deposits.

- Fuzzy Analytic Hierarchy Process (FAHP)

Our approach begins with the FAHP, which combines fuzzy logic with the traditional Analytic Hierarchy Process (AHP) to handle the inherent uncertainties in evaluating multiple criteria. This process starts by assigning scores to various natural and anthropogenic factors such as natural reserves, water reservoirs, and land use. These factors are categorized under broader evaluation criteria like storage capacity, accessibility, and energy consumption.

The FAHP enhances the AHP by incorporating fuzzy member functions, which allow for the expression of vagueness and imprecision in human judgment^89^. We then normalize the assigned weights using FAHP, ensuring consistency across all criteria.

Subsequently, AHP's pairwise comparison matrix calculates the relative weights of these criteria, addressing both the importance and the interdependency among them. The integration of these weighted criteria through Geographic Information System (GIS) analysis, along with fuzzy functions, creates an overlay of layers that form the basis for further analysis by ML algorithms.

To refine the suitability map, we employed various ML algorithms: CatBoost, Gradient Boosting Tree (GBT), k-Nearest Neighbors (KNN), Light Gradient Boosting Machine (LGBM), Multilayer Perceptron (MLP), Logistic Regression (LR), Support Vector Regression (SVR), and XGBoost. These algorithms were trained using 70% of the collected data, with the remaining 30% split equally for validation and testing purposes.

- Performance Metrics

The selected ML algorithms were trained and tested on this dataset, and their performance was evaluated using Mean Squared Error (MSE), Mean Absolute Error (MAE), Mean Absolute Percentage Error (MAPE), Root Mean Square Error (RMSE), and Correlation Coefficient (R^2^). These metrics (Eqs. 1–5)^17,90,91^ were crucial in assessing the algorithms' effectiveness and ensuring the model's accuracy before applying it to the entire dataset. The ultimate goal was to identify optimal locations for UHS .

1 $$\text{MSE}=\frac{1}{\text{N}}\sum_{\text{i}=1}^{\text{N}} {\left({\text{y}}_{\text{i}}-{\widehat{\text{y}}}_{\text{i}}\right)}^{2}$$

2 $$\text{MAE}=\frac{1}{\text{N}}\sum_{\text{i}=1}^{\text{N}} \left|{\text{y}}_{\text{i}}-{\widehat{\text{y}}}_{\text{i}}\right|$$

3 $$\text{MAPE}=\frac{1}{\text{N}}\sum_{\text{i}=1}^{\text{N}} \left|\frac{{\text{y}}_{\text{i}}-{\widehat{\text{y}}}_{\text{i}}}{{\text{y}}_{\text{i}}}\right|$$

4 $${\text{RMSE}} = \sqrt {\frac{1}{N}\mathop \sum \limits_{i = 1}^{{\text{N}}} \left( {{\text{y}}_{{\text{i}}} - {\hat{\text{y}}}_{{\text{i}}} } \right)^{2} }$$

5 $${\text{R}}^{2}=1-\frac{\sum_{\text{i}} {\left({\text{y}}_{\text{i}}-{\widehat{\text{y}}}_{\text{i}}\right)}^{2}}{\sum_{\text{i}} {\left({\text{y}}_{\text{i}}-\overline{\text{y} }\right)}^{2}}$$

where y_i_ is the ith observed value, ${\widehat{\text{y}}}_{\text{i}}$ is the corresponding predicted value for y_i_, and n is the number of observations.

### Data preprocessing and analysis

The data are required to be preprocessed before uploading to any ML model. The preprocessing includes multiple data transformation steps such as data resampling, standardization or normalization, noise elimination, outlier removal, etc^77^. These steps aid to enhance the forecasting accuracy of data-driven algorithms.

After clearing data, the first step is normalization, and Eq. 6 is used for this purpose.

6 $${X}_{N}= \frac{\left({X}_{R}-{X}_{\text{minimum }}\right)}{\left({X}_{\text{maximum }}-{X}_{\text{minimum}}\right)}$$

Here, X_N_ represents the normalized value, X_R_ is the value to be normalized, X_minimum_ is the minimum value in all the values for related variables, and X_maximum_ is the maximum value in all the values for related variables^92^.

**Figure 3:**
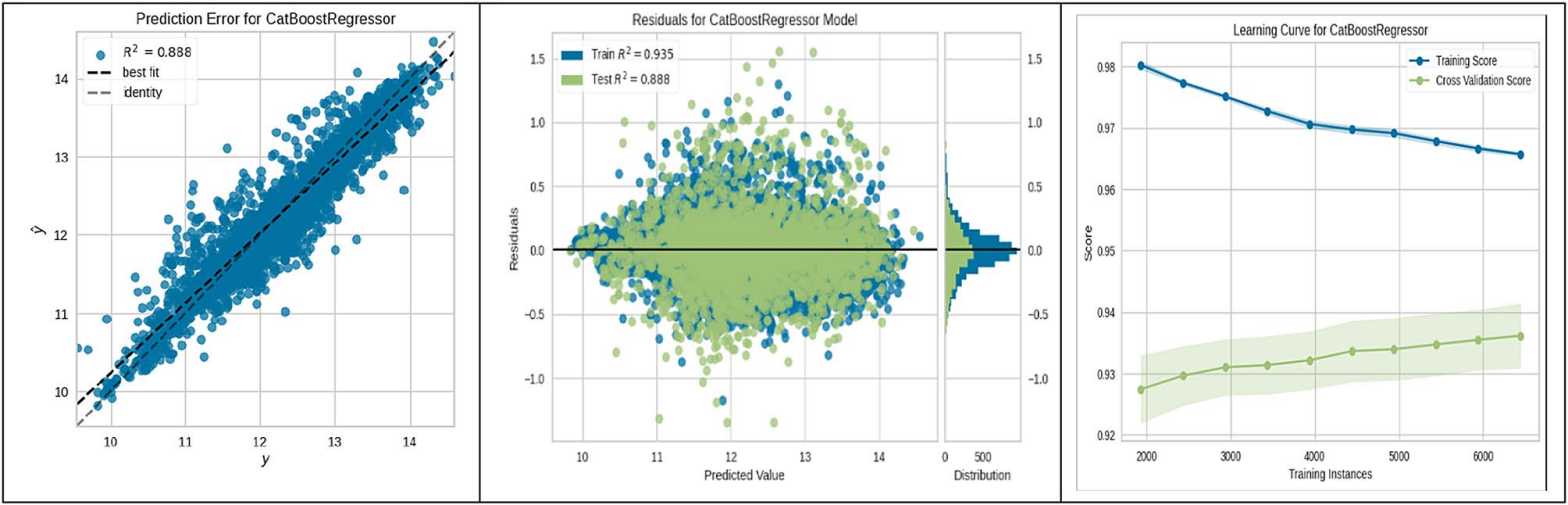
CatBoost performance.

**Figure 4:**
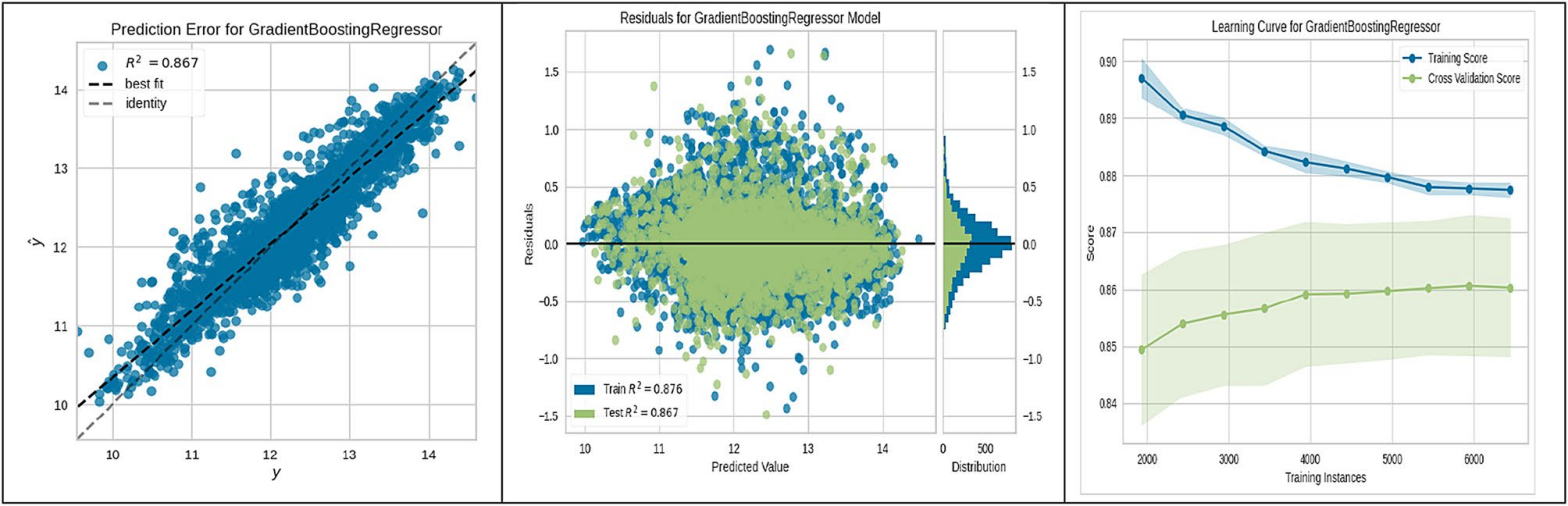
GBT performance.

**Figure 5:**
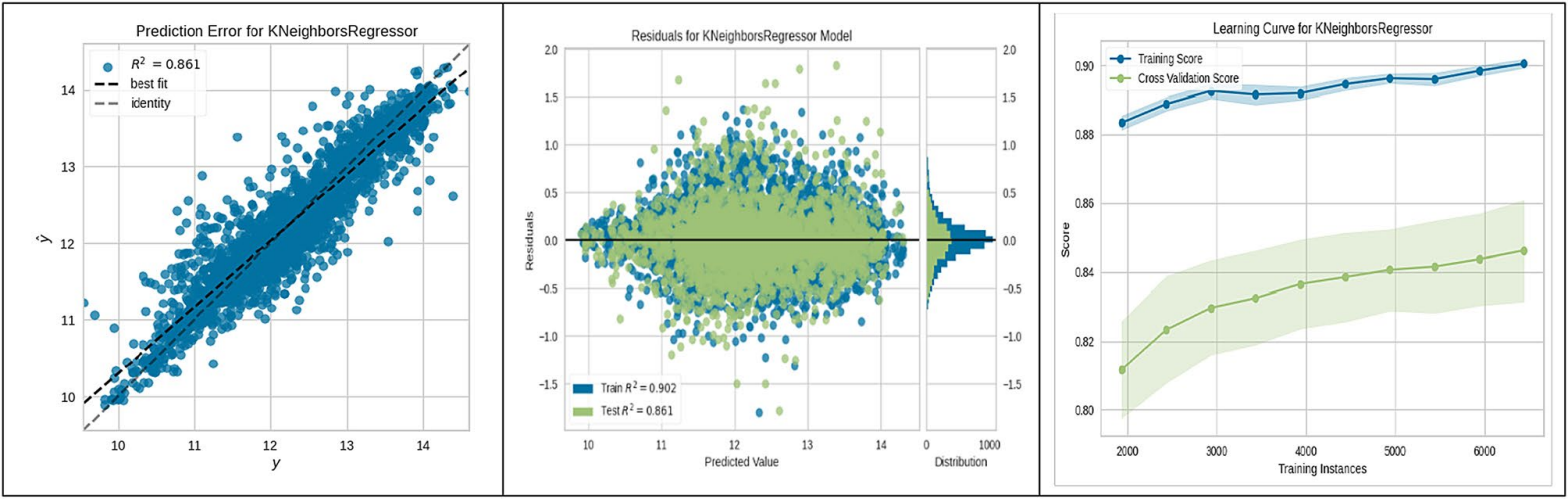
KNN performance.

**Figure 6:**
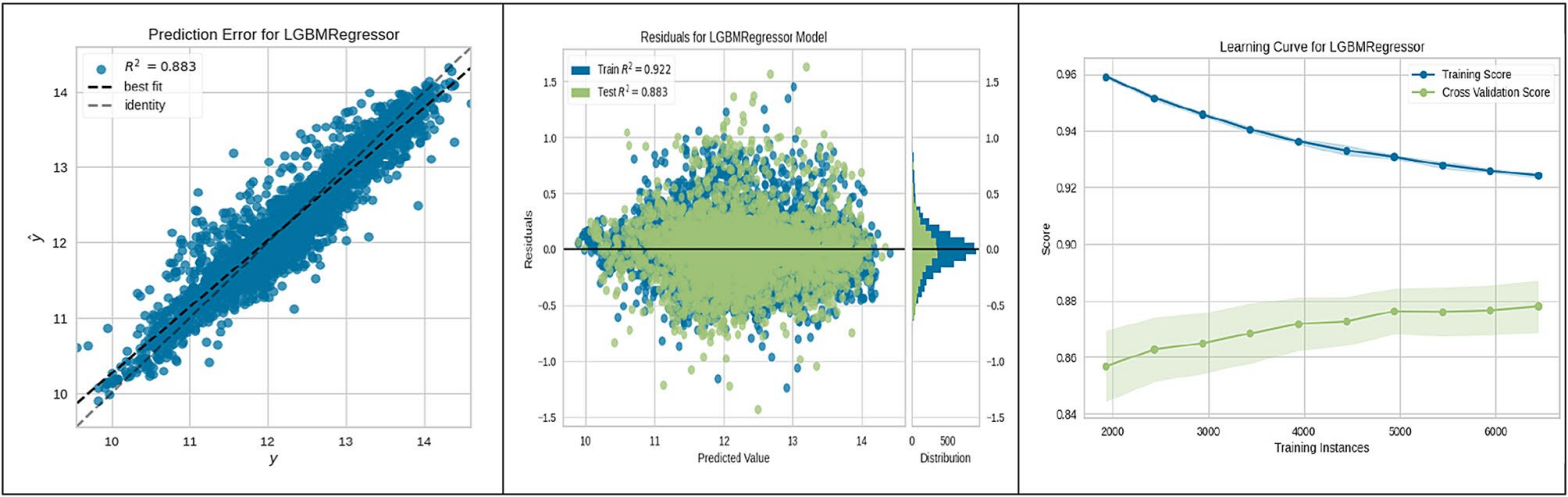
LGBM performance.

**Figure 7:**
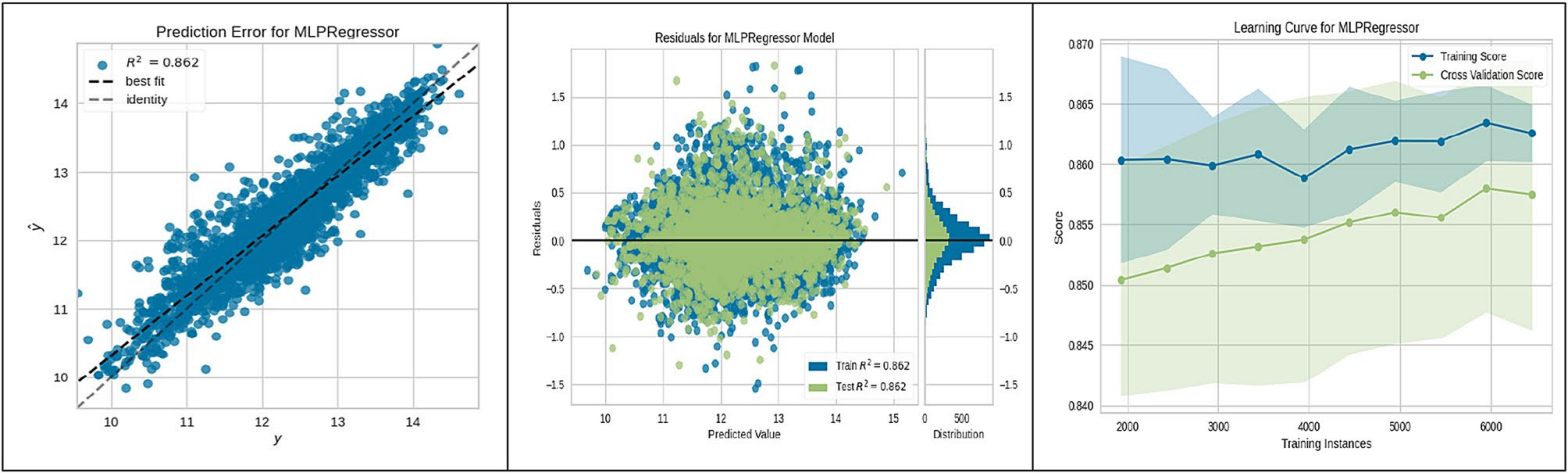
MLP performance.

**Figure 8:**
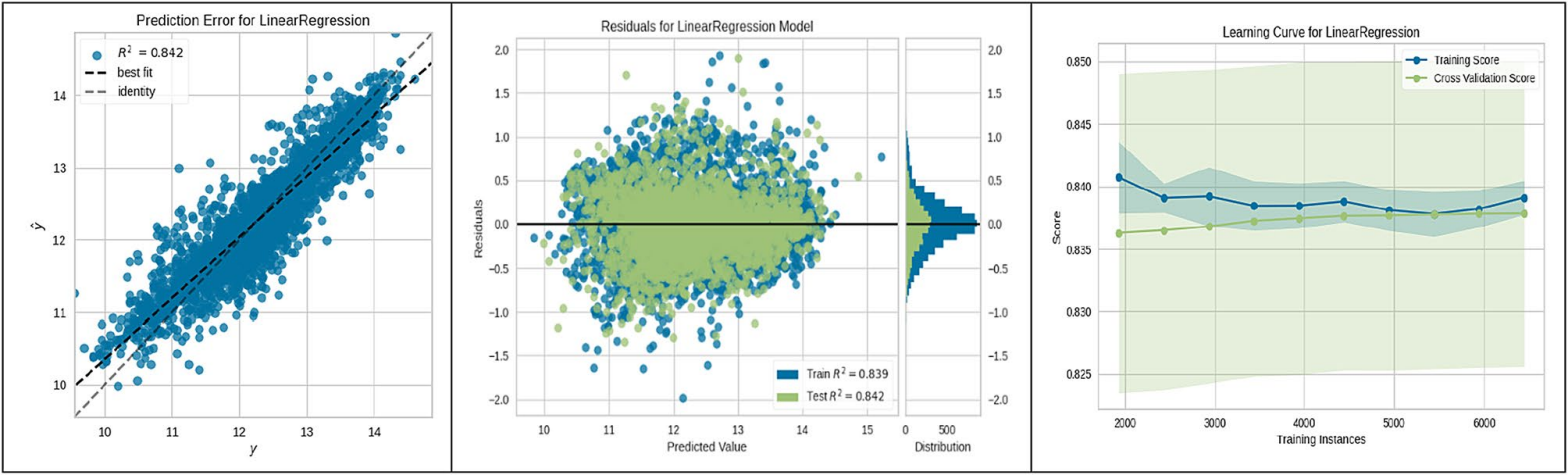
LR performance.

**Figure 9:**
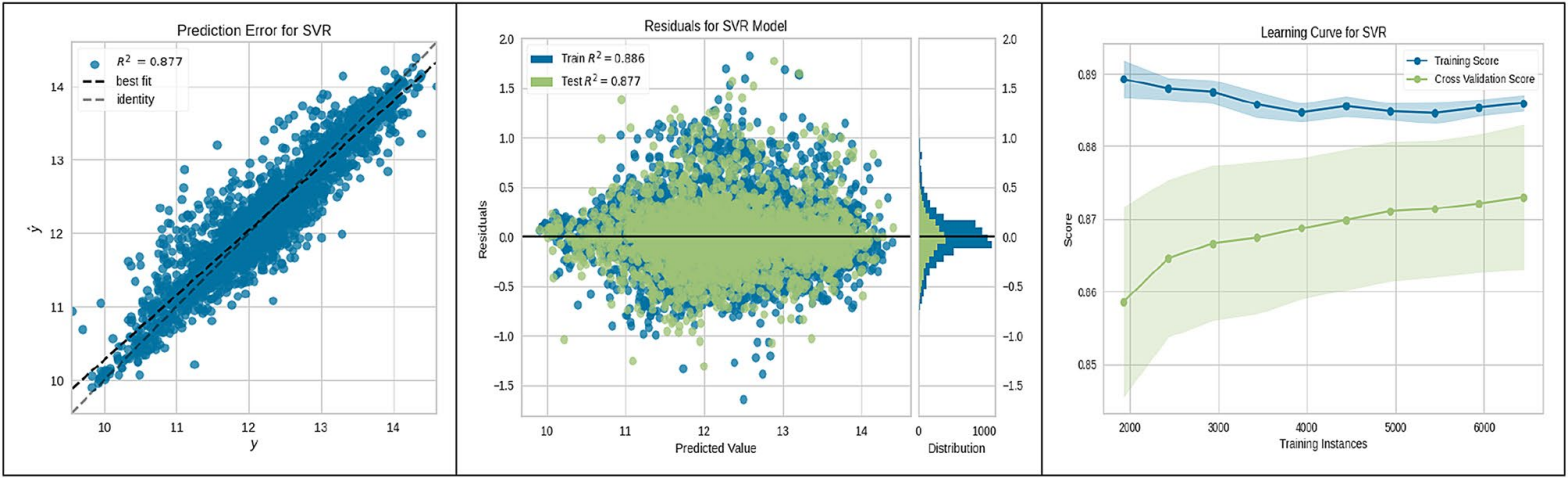
SVR performance.

**Figure 10:**
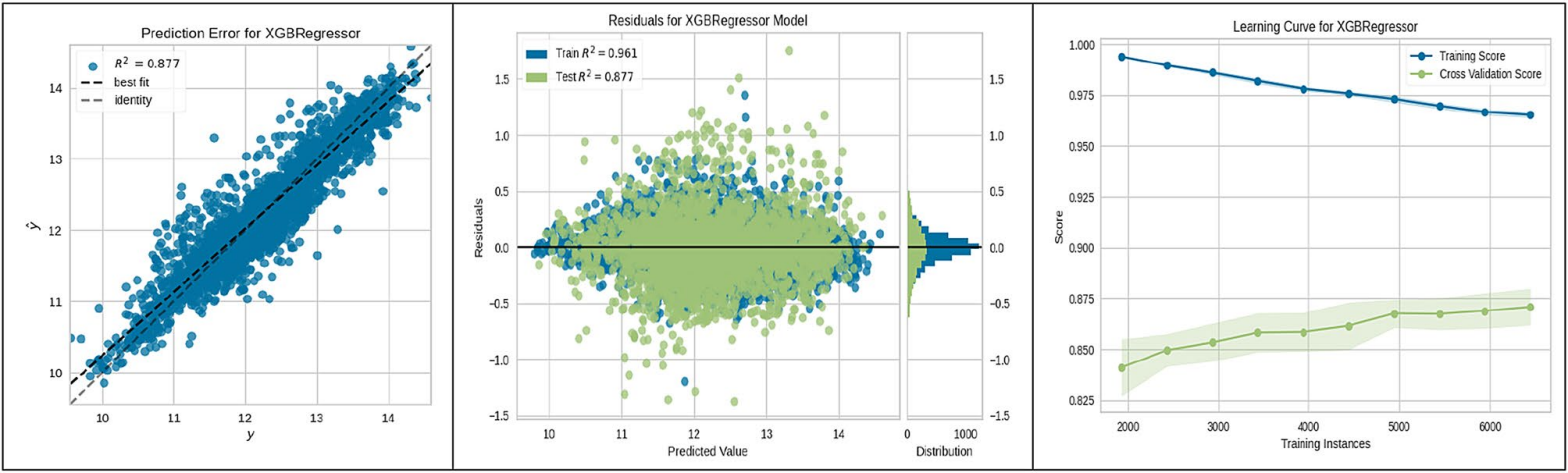
XGBoost performance.

In evaluating machine-learning algorithms for predicting the suitability of sites for underground hydrogen storage in Poland, our findings are depicted in Figs. 3, 4, 5, 6, 7, 8, 9 and 10. Each figure provides a comprehensive overview of the algorithm's performance, showcasing the strong predictive accuracy and reliability of the models used. The CatBoost Regressor, as shown in Fig. 3, demonstrated exceptional performance with a high correlation between observed and predicted values (R^2^ = 0.888). This strong correlation was supported by a consistent R^2^ value for both training and test data and a learning curve indicating the model's ability to generalize without overfitting.

Figure 4 illustrates the Gradient Boosting Regressor's robust performance with a solid R^2^ of 0.867. The model's residuals and learning curve further suggest stable performance and good generalization capabilities, even as the number of training instances increases.

The K-Nearest Neighbours (KNN) algorithm, analysed in Fig. 5, also showed significant prediction accuracy (R^2^ = 0.861). The consistency of its training score and the improvement in the cross-validation score with additional data indicate its effectiveness in learning from the increasing dataset.

The Light Gradient Boosting Machine (LGBM) Regressor, discussed in Fig. 6, displayed a strong correlation between observed and predicted values (R^2^ = 0.883) and a learning curve demonstrating the model's growing accuracy with more data points.

Figure 7 highlighted the performance of the Multilayer Perceptron (MLP) Regressor, revealing a reliable predictive accuracy (R^2^ = 0.862) and a stable performance across varying sizes of training data, suggesting the model's proficiency in learning effectively.

Linear Regression (LR) model efficacy, represented in Fig. 8, confirmed a strong linear relationship and predictive capability (R^2^ = 0.842). The model's learning curve indicates consistent and generalizable performance throughout training. Support Vector Regression (SVR), shown in Fig. 9, achieved a strong predictive accuracy (R^2^ = 0.877) with a learning curve reflecting a positive performance trajectory as more data is introduced, underscoring the model's generalization strength. Lastly, the XGBoost Regressor, detailed in Fig. 10, exhibited high predictive accuracy (R^2^ = 0.877) and a learning curve suggestive of robust learning ability, with an upward trend in cross-validation scores as the number of training instances expanded.

These figures substantiate the high accuracy and generalization capabilities of the ML algorithms employed, with the CatBoost model being particularly noteworthy for its superior performance. This empirical observation from the CatBoost model's analysis has yielded actionable insights into site suitability for UHS. Such contributions are significant to the research field, laying a strong groundwork for enhancing the methodologies used in future site selection and policy planning.

Table 1 summarizes the performance of various machine learning algorithms evaluated based on four key metrics: Mean Absolute Error (MAE), Mean Squared Error (MSE), Root Mean Square Error (RMSE), and Mean Absolute Percentage Error (MAPE). The algorithms assessed include CatBoost, LightGBM (Lgbm), XGBoost, Gradient Boosting Regressor (Gbr), K-Nearest Neighbors (Knn), Linear Regression (Lr), Support Vector Regression (Svr), and Multilayer Perceptron (MLP). The results indicate that CatBoost outperforms other algorithms across all metrics, suggesting its superior predictive capability within the evaluated dataset.

**Table 1:** Comparative performance metrics of machine learning algorithms for predictive modelling.

		Criteria			
		MAE	MSE	RMSE	MAPE
ML Algorithms	Catboost	0.1994	0.0816	0.2833	0.0163
	Lgbm	0.2102	0.0866	0.2920	0.0172
	XGboost	0.2152	0.0911	0.2996	0.0176
	Gbr	0.2285	0.0995	0.3124	0.0187
	Knn	0.2333	0.1088	0.3274	0.0191
	Lr	0.2461	0.1159	0.3372	0.0202
	Svr	0.2065	0.0905	0.2982	0.0169
	MLP	0.2322	0.1032	0.3186	0.0190

In assessing the suitability of the Na1 rock salt deposit in the Fore-Sudetic Monocline for underground hydrogen storage, our research meticulously compared machine-learning algorithms, concluding that CatBoost outperforms its counterparts. It achieved the most favourable error metrics, with an MAE of 0.1994, MSE of 0.0816, RMSE of 0.2833, and a notably low MAPE of 0.0163, underscoring its precision in predictive modelling. Other evaluated algorithms, namely SVR, MLP, KNN, and XGBoost, yielded higher errors, with MAE values ranging from 0.2065 to 0.2461, MSE values from 0.0905 to 0.1159, RMSE values from 0.2982 to 0.3372, and MAPE values from 0.0169 to 0.0202. Given these results, CatBoost is identified as the most reliable algorithm for forecasting the suitability of salt caverns for hydrogen storage in the geological context of Poland.

**Figure 11:**
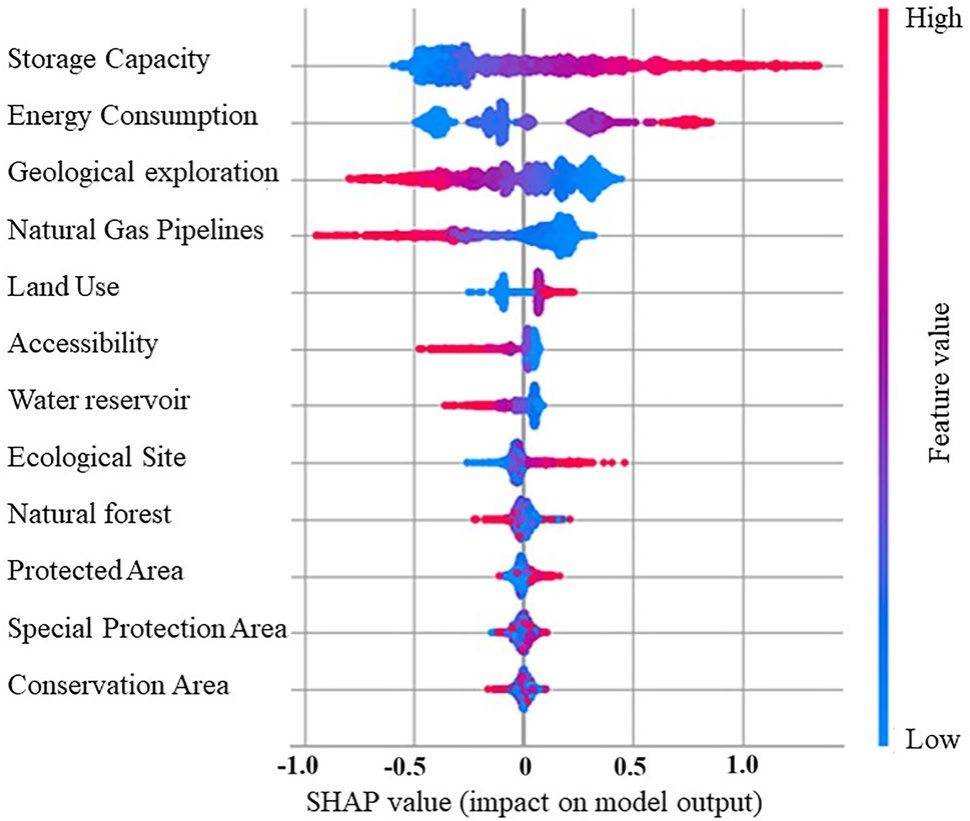
Feature importance of CatBoost model.

Another point worth mentioning is the good trend of SHAP values illustrated in Fig. 11. The clear trend of SHAP (Shapley Additive Explanations) values highlights the advantage of SHAP as a model-agnostic tool for feature importance analysis. Grounded in game theory, SHAP values estimate each feature's contribution to the model's prediction. This model-agnostic approach allows for examining underlying patterns using ML/AI models without the constraint of assuming perfect model representation. Consequently, it mitigates interpretative bias, offering a more robust understanding of feature impact. The SHAP Value Summary Plot depicted in Fig. 11 demonstrates the influence of each feature on the model's output: features are colour-coded (with red indicating high and blue indicating low values) to show their impact on predictions. Features like Storage Capacity and Energy Consumption have a significant impact, marking them as key determinants for assessing the viability of salt caverns for hydrogen storage. In contrast, distances to Conservation Areas and Special Protection Areas display variable impacts, indicating their different levels of influence on the model's predictions across the dataset.

## Results and discussion

The seminal work by Lankof and Tarkowski^76^ provides a robust foundation for site selection methodology using multi-criteria decision analysis and GIS. Their approach represents a significant step in identifying suitable locations for hydrogen storage within bedded salt deposits. Our present study builds upon this foundation and introduces an innovative artificial intelligence (AI) framework that enhances the site-selection process.

While Lankof and Tarkowski^76^ focus on the application of a multi-criteria decision analysis in a GIS setting, the present study expands this by incorporating a suite of eight AI algorithms. This inclusion goes beyond the traditional GIS analyses by enabling a data-driven, machine-learning approach that offers increased accuracy and computational efficiency. Notably, our work emphasizes the superior performance of the CatBoost algorithm in evaluating the suitability of salt caverns for hydrogen storage, which complements and quantitatively surpasses the earlier methodologies.

Furthermore, our research provides a comprehensive comparison between traditional methods, such as the Analytic Hierarchy Process (AHP), and advanced machine learning techniques, showcasing the latter's enhanced capabilities in creating detailed suitability maps. This methodological advancement is crucial for stakeholders involved in the strategic development of underground hydrogen storage facilities. By employing AI algorithms, the present study presents a cutting-edge methodology that can inform decision-making for governmental bodies, geological services, and the renewable energy industry.

Moreover, our results contribute to the ongoing scientific discourse on underground hydrogen storage by offering empirical evidence of the effectiveness of AI in the site selection process. The adaptability of our AI framework underscores its potential application on an international scale, supporting the strategic infrastructure development for renewable energy storage. So, our research not only aligns with the objectives of Lankof and Tarkowski's work^76^ but also extends it by leveraging the latest advancements in AI, thereby providing a novel and empirically validated approach to the selection of underground hydrogen storage sites.

This research advances the application of an artificial intelligence (AI) approach to strategically selecting prime locations for Underground Hydrogen Storage (UHS) within bedded rock salt formations. Historically, multi-criteria decision analysis has been harnessed in site-selection studies, particularly for evaluating distinct salt structures for hydrogen storage. However, the dedicated application of AI algorithms for identifying optimal UHS sites in bedded rock salt deposits is a novel exploration presented within this paper.

Machine Learning (ML) methods, which utilize algorithms to learn from and make inferences from data, are employed herein without explicit programming. Concurrently, the Analytic Hierarchy Process (AHP) is utilized to assign relative importance to various criteria, a technique especially useful in morphometric analysis of watersheds where quantification of factors like rainfall or soil characteristics may be imprecise.

Despite their respective advantages, both AHP and ML methodologies are subject to practical limitations. AHP's reliance on expert judgment for rule definition can result in difficult models to interpret and validate. Conversely, ML's efficacy is tethered to the quality and volume of the data, as well as the algorithm and parameter selections, which, if not judiciously chosen, can lead to overfitting or underfitting, thereby diminishing the model's predictive capability on novel data sets.

The present study divided the data into two subsets: a training set constituting 70% of the total data, and a validation-testing set forming the remaining 30%. The performance of various ML algorithms was rigorously evaluated on both the training and testing datasets, as illustrated in Figs. 3, 4, 5, 6, 7, 8, 9 and 10. These figures juxtapose the target values derived from AHP against the predictions made by the algorithms, thereby calculating the error of the models. The numerical outcomes were closely aligned with those procured from the KNN, SVM, LightGBM, XGBoost, MLP, CatBoost, GBR, and MLR methods, as presented in Table 1. The CatBoost model, in particular, exhibited enhanced performance compared to its counterparts.

**Figure 12:**
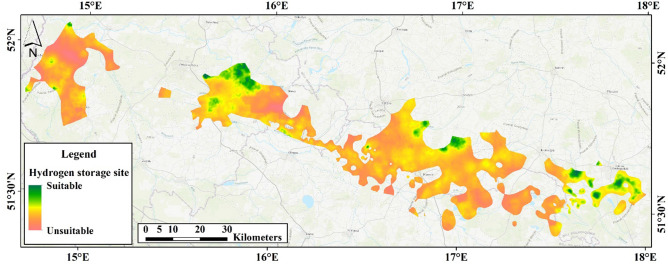
Map displaying the outcomes obtained through the implementation of the optimal machine-learning algorithm, delineating potential sites for hydrogen storage within the region, generated using ArcGIS Pro 2.8 software. The base map was developed by Esri using HERE data, DeLorme base map layers, OpenStreetMap contributors, Esri base map data, and select data from the GIS user community. For more information about Esri® software, please visit http://www.esri.com.

Twelve input data layers were processed through the AI algorithm to identify suitable UHS locations within Poland, Conservation Area, Geological exploration, Water reservoir, Accessibility, Ecological Site, Energy Consumption, Land Use, Natural Gas Pipelines, Natural forest, Protected Area, Special Protection Area, Storage Capacity. After selecting the optimal method from the suite of evaluated ML algorithms, the chosen model was applied to the entire study area (Fig. 12). The resulting performance was assessed against the outcomes derived from the AHP technique, with the ML model demonstrating greater accuracy and computational efficiency than the AHP model, thereby solidifying the potential of AI in streamlining UHS site selection.

Our research demonstrates that the selected methodology markedly impacts the generated suitability maps, proving an efficient instrument for swiftly pinpointing optimal locations for Underground Hydrogen Storage (UHS). The comparison of the spatial output derived from AI algorithms with the findings of Lankof and Tarkowski^76^ validates the commendable accuracy of the algorithms utilized^88^.

The present study's results, as depicted in Fig. 12, illustrate the sites deemed suitable for Underground Hydrogen Storage (UHS) within Poland, which were identified through the application of advanced machine learning (ML) algorithms. A comparison of Lankof & Tarkowski's research^76^ shows some discrepancies in the potential sites across different regions in Poland.

We utilized a robust dataset divided into a training set, constituting 70% of the total data, and a validation-testing set for the remaining 30%. The ML algorithms were not only trained on this dataset but also rigorously tested and validated to ensure the generalizability of the predictions. The performance metrics, thoroughly detailed in Table 1, reflect the algorithms' accuracy and predictive quality. Specifically, the CatBoost model exhibited superior performance, underlined by its high precision in mapping the complex interrelations of the criteria defining the suitability for UHS.

The variations in suitable sites between the studies can be attributed to the diverse analytical mechanisms intrinsic to different ML algorithms compared to the GIS-based MCDA employed by Lankof and Tarkowski^76^. The ML approach takes into account a broader range of factors and their interactions, allowing for the identification of patterns that may not be apparent through traditional methods.

To further expound on the results obtained from our AI algorithms, we have delved into a feature importance analysis. This analysis, using techniques such as SHAP (Shapley Additive explanations), clarifies the contribution of each criterion to the predictive models. This step is crucial for understanding how specific factors such as conservation areas, geological exploration, and energy consumption significantly influence the algorithms' output, thereby demystifying the ML process. By conducting this comprehensive analysis and comparison, we demonstrate the efficacy and accuracy of ML algorithms in identifying suitable UHS locations. This demonstrates that our selected methodology can successfully supplement and potentially improve upon traditional approaches, providing an efficient means for swiftly identifying prime locations for UHS. The data were rendered into a raster map, culminating in a final visualization that illustrates the potential of various locations for UHS. This suitability map clearly delineates areas within the rock salt strata that hold promise for hydrogen storage, allowing for straightforward identification of prospective sites. The most favourable sites—characterized by high storage capacity and favourable ratings across all assessed criteria—are predominantly located in the central-western segment of the study area. Furthermore, the map differentiates areas of high suitability based on a composite of criteria. The most advantageous areas in the monocline's western regions are those with substantial storage volumes and extensive geological investigation. Conversely, in the eastern sectors of the surveyed region, high suitability correlates with factors such as elevated energy demand, the extent of geological exploration, and proximity to existing gas pipeline infrastructure.

## Conclusion

This study systematically applied eight artificial intelligence algorithms—namely KNN, SVM, LightGBM, XGBoost, MLP, CatBoost, GBR, and MLR—to scout for viable underground hydrogen storage (UHS) locations within Poland. The research established a robust AI-informed framework by leveraging a multifaceted dataset comprising storage capacity, proximity to water sources, transportation networks, pipelines, boreholes, energy consumption, and land use. Our comparative analysis pinpointed the CatBoost algorithm as the most precise tool for delineating favourable UHS sites within the rock salt strata, offering an accurate numerical assessment of their potential. The efficacy of the machine learning approach was benchmarked against the Analytic Hierarchy Process (AHP), with CatBoost demonstrating enhanced accuracy and computational efficiency. These advancements present actionable intelligence and novel strategic avenues for stakeholders, including policy planners, geological services, renewable energy producers, and entities within the chemical and petrochemical sectors, who are invested in the strategic development of UHS facilities. The implications of our work extend to governmental and European Union institutions, which are key players in the infrastructure development for renewable energy storage. Additionally, the outcomes of this research are poised to contribute significantly to the ongoing discourse within the scientific community regarding hydrogen storage solutions, offering empirical data to inform policy decisions. The adaptability of the proposed AI methodology underscores its potential for broader international application in selecting sites for underground energy storage, subject to region-specific modifications and criteria. Future research directions include conducting comparative analyses of these contemporary AI methodologies against traditional site selection practices. Such studies would be instrumental in identifying new, sustainable UHS sites, further streamlining the site selection process, enhancing operational efficiency, and ensuring the conservation of time and resources in future UHS ventures.

### Supplementary Information

Supplementary Information.

## Data Availability

The code and the datasets generated and analyzed during the current study would be available on reasonable request.

## References

[1] Osman AI (2022). Hydrogen production, storage, utilisation and environmental impacts: A review. Environ. Chem. Lett..

[2] Garcia DA, Barbanera F, Cumo F, Di Matteo U, Nastasi B (2016). Expert opinion analysis on renewable hydrogen storage systems potential in Europe. Energies (Basel).

[3] Ishaq H, Dincer I, Crawford C (2022). A review on hydrogen production and utilization: Challenges and opportunities. Int. J. Hydrog. Energy.

[4] Zhang F, Zhao P, Niu M, Maddy J (2016). The survey of key technologies in hydrogen energy storage. Int. J. Hydrog. Energy.

[5] El-Shafie M, Kambara S, Hayakawa Y (2019). Hydrogen production technologies overview. J. Power Energy Eng..

[6] Abdalla AM (2018). Hydrogen production, storage, transportation and key challenges with applications: A review. Energy Convers. Manag..

[7] Tarkowski R, Lankof L, Luboń K, Michalski J (2024). Hydrogen storage capacity of salt caverns and deep aquifers versus demand for hydrogen storage: A case study of Poland. Appl. Energy.

[8] Andersson J, Grönkvist S (2019). Large-scale storage of hydrogen. Int. J. Hydrog. Energy.

[9] Hassan IA, Ramadan HS, Saleh MA, Hissel D (2021). Hydrogen storage technologies for stationary and mobile applications: Review, analysis and perspectives. Renew. Sustain. Energy Rev..

[10] Bünger, U., Michalski, J., Crotogino, F. & Kruck, O. Large-scale underground storage of hydrogen for the grid integration of renewable energy and other applications. in *Compendium of Hydrogen Energy: Hydrogen Use, Safety and the Hydrogen Economy*, vol. 4 (2015).

[11] Crotogino, F., Donadei, S., Bünger, U., & Landinger, H. Large-scale hydrogen underground storage for securing future energy supplies. in *18th World Hydrogen Energy Conference 2010 - WHEC 2010 Parallel Sessions Book 4: Storage Systems / Policy Perspectives, Initiatives and Co-operations* 78, (2010).

[12] Lankof L, Luboń K, Le Gallo Y, Tarkowski R (2024). The ranking of geological structures in deep aquifers of the Polish Lowlands for underground hydrogen storage. Int. J. Hydrog. Energy.

[13] Bui DT (2018). Land subsidence susceptibility mapping in South Korea using machine learning algorithms. Sensors (Switzerland).

[14] Corsini A, Cervi F, Ronchetti F (2009). Weight of evidence and artificial neural networks for potential groundwater spring mapping: An application to the Mt. Modino area (Northern Apennines, Italy). Geomorphology.

[15] Naghibi SA, Pourghasemi HR (2015). A comparative assessment between three machine learning models and their performance comparison by bivariate and multivariate statistical methods in groundwater potential mapping. Water Resour. Manag..

[16] Arabameri A (2020). Landslide susceptibility evaluation and management using different machine learning methods in the Gallicash River Watershed Iran. Remote Sens. (Basel).

[17] Derakhshani R (2023). Machine learning-based assessment of watershed morphometry in Makran. Land (Basel).

[18] Tewari, S. Assessment of data-driven ensemble methods for conserving wellbore stability in deviated wells. in *Proceedings - SPE Annual Technical Conference and Exhibition* vols 2019 (2019).

[19] Tariq Z (2023). Enhancing wettability prediction in the presence of organics for hydrogen geo-storage through data-driven machine learning modeling of rock/H2/brine systems. Fuel.

[20] Zhang H (2023). Improving predictions of shale wettability using advanced machine learning techniques and nature-inspired methods: Implications for carbon capture utilization and storage. Sci. Total Environ..

[21] Kohzadvand K, Kouhi MM, Barati A, Omrani S, Ghasemi M (2023). Prediction of interfacial wetting behavior of H2/mineral/brine; implications for H2 geo-storage. J. Energy Storage.

[22] Behnamnia M, Mozafari N, Dehghan Monfared A (2023). Rigorous hybrid machine learning approaches for interfacial tension modeling in brine-hydrogen/cushion gas systems: Implication for hydrogen geo-storage in the presence of cushion gas. J. Energy Storage.

[23] Gbadamosi A (2024). New-generation machine learning models as prediction tools for modeling interfacial tension of hydrogen-brine system. Int. J. Hydrog. Energy.

[24] Hosseini M, Leonenko Y (2024). Prediction of hydrogen−brine interfacial tension at subsurface conditions: Implications for hydrogen geo-storage. Int. J. Hydrog. Energy.

[25] Ng CSW, Djema H, Nait Amar M, Jahanbani Ghahfarokhi A (2022). Modeling interfacial tension of the hydrogen-brine system using robust machine learning techniques: Implication for underground hydrogen storage. Int. J. Hydrog. Energy.

[26] Omrani S (2023). Interfacial tension-temperature-pressure-salinity relationship for the hydrogen-brine system under reservoir conditions: Integration of molecular dynamics and machine learning. Langmuir.

[27] Ansari S (2022). Prediction of hydrogen solubility in aqueous solutions: Comparison of equations of state and advanced machine learning-metaheuristic approaches. Int. J. Hydrog. Energy.

[28] Tatar A, Esmaeili-Jaghdan Z, Shokrollahi A, Zeinijahromi A (2022). Hydrogen solubility in n-alkanes: Data mining and modelling with machine learning approach. Int. J. Hydrog. Energy.

[29] Vo Thanh H (2024). Data-driven machine learning models for the prediction of hydrogen solubility in aqueous systems of varying salinity: Implications for underground hydrogen storage. Int. J. Hydrog. Energy.

[30] Zhang J, Clennell MB, Sagotra A, Pascual R (2023). Molecular dynamics simulation and machine learning for predicting hydrogen solubility in water: Effects of temperature, pressure, finite system size and choice of molecular force fields. Chem. Phys..

[31] Li J (2022). Machine-learning-based capacity prediction and construction parameter optimization for energy storage salt caverns. Energy.

[32] Kanaani M, Sedaee B, Asadian-Pakfar M, Gilavand M, Almahmoudi Z (2023). Development of multi-objective co-optimization framework for underground hydrogen storage and carbon dioxide storage using machine learning algorithms. J. Clean Prod..

[33] Elabbassi I, Khala M, Elyanboiy N, Eloutassi O, El hassouani Y (2024). Evaluating and comparing machine learning approaches for effective decision making in renewable microgrid systems. Results Eng..

[34] Mubarak Y, Koeshidayatullah A (2023). Hierarchical automated machine learning (AutoML) for advanced unconventional reservoir characterization. Sci. Rep..

[35] Soltanian MR (2024). Data driven simulations for accurately predicting thermodynamic properties of H2 during geological storage. Fuel.

[36] Zivar D, Kumar S, Foroozesh J (2021). Underground hydrogen storage: A comprehensive review. Int. J. Hydrog. Energy.

[37] Tarkowski R (2019). Underground hydrogen storage: Characteristics and prospects. Renew. Sustain. Energy Rev..

[38] Heinemann N (2021). Enabling large-scale hydrogen storage in porous media-the scientific challenges. Energy Environ. Sci..

[39] Sambo C (2022). A review on worldwide underground hydrogen storage operating and potential fields. Int. J. Hydrog. Energy.

[40] Aftab A, Hassanpouryouzband A, Xie Q, Machuca LL, Sarmadivaleh M (2022). Toward a fundamental understanding of geological hydrogen storage. Ind. Eng. Chem. Res..

[41] Thiyagarajan SR, Emadi H, Hussain A, Patange P, Watson M (2022). A comprehensive review of the mechanisms and efficiency of underground hydrogen storage. J. Energy Storage.

[42] Navaid HB, Emadi H, Watson M (2023). A comprehensive literature review on the challenges associated with underground hydrogen storage. Int. J. Hydrog. Energy.

[43] Acht, A. & Donadei, S. *Hydrogen Storage in Salt Caverns: State of the Art, New Developments and R&D Projects*. SMRI Fall 2012 Technical Conference (2012).

[44] Kruck, O., Crotogino, F., Prelicz, R. & Rudolph, T. *Overview on all known Underground Storage Technologies for Hydrogen*. HyUnder (2013).

[45] Tarkowski R, Czapowski G (2018). Salt domes in Poland—potential sites for hydrogen storage in caverns. Int. J. Hydrog. Energy.

[46] Muhammed NS (2022). A review on underground hydrogen storage: Insight into geological sites, influencing factors and future outlook. Energy Rep..

[47] Hevin, G. Underground storage of Hydrogen in salt caverns. In *Proceedings of the European Workshop on Underground Energy Storage, Paris, France* 7–8 (2019).

[48] Basniev, K. S., Omelchenko, R. J. & Adzynova, F. A. Underground hydrogen storage problems in Russia. In *18th World Hydrogen Energy Conference 2010* (2010).

[49] Ponomarev-Stepnoi NN (2004). Nuclear-hydrogen power. Atomic Energy.

[50] Raza A (2022). A holistic overview of underground hydrogen storage: Influencing factors, current understanding, and outlook. Fuel.

[51] Liu W (2020). Feasibility evaluation of large-scale underground hydrogen storage in bedded salt rocks of China: A case study in Jiangsu province. Energy.

[52] Caglayan DG (2020). Technical potential of salt caverns for hydrogen storage in Europe. Int. J. Hydrog. Energy.

[53] Lankof L, Tarkowski R (2020). Assessment of the potential for underground hydrogen storage in bedded salt formation. Int. J. Hydrog. Energy.

[54] Williams JDO (2022). Does the United Kingdom have sufficient geological storage capacity to support a hydrogen economy? Estimating the salt cavern storage potential of bedded halite formations. J. Energy Storage.

[55] Lankof L, Urbańczyk K, Tarkowski R (2022). Assessment of the potential for underground hydrogen storage in salt domes. Renew. Sustain. Energy Rev..

[56] Chen F (2023). Capacity assessment and cost analysis of geologic storage of hydrogen: A case study in Intermountain-West Region USA. Int. J. Hydrog. Energy.

[57] Lankof L, Nagy S, Polański K, Urbańczyk K (2022). Potential for underground storage of liquid fuels in bedded rock salt formations in Poland. Energies (Basel).

[58] Ślizowski J, Lankof L, Urbańczyk K, Serbin K (2017). Potential capacity of gas storage caverns in rock salt bedded deposits in Poland. J. Nat. Gas Sci. Eng..

[59] Cyran K, Kowalski M (2021). Shape modelling and volume optimisation of salt caverns for energy storage. Appl. Sci. (Switzerland).

[60] Bérest P, Bergues J, Brouard B (1999). Review of static and dynamic compressibility issues relating to deep underground salt caverns. Int. J. Rock Mech. Min. Sci..

[61] Wang T (2013). A new shape design method of salt cavern used as underground gas storage. Appl. Energy.

[62] Böttcher N, Görke UJ, Kolditz O, Nagel T (2017). Thermo-mechanical investigation of salt caverns for short-term hydrogen storage. Environ. Earth Sci..

[63] Bérest P, Louvet F (2020). Aspects of the thermodynamic behavior of salt caverns used for gas storage. Oil Gas Sci. Technol..

[64] Cyran K (2020). Insight into a shape of salt storage caverns. Arch. Min. Sci..

[65] Matos CR, Carneiro JF, Pereira da Silva P, Henriques CO (2021). A GIS-MCDA approach addressing economic-social-environmental concerns for selecting the most suitable compressed air energy storage reservoirs. Energies (Basel).

[66] Cai B (2017). Environmental concern-based site screening of carbon dioxide geological storage in China. Sci. Rep..

[67] Roberts-Ashby T, Ashby B (2016). A method for examining the geospatial distribution of CO2 storage resources applied to the Pre-Punta Gorda composite and Dollar Bay reservoirs of the South Florida Basin, U.S.A. Mar. Pet. Geol..

[68] Parkes D, Evans DJ, Williamson P, Williams JDO (2018). Estimating available salt volume for potential CAES development: A case study using the Northwich Halite of the Cheshire Basin. J. Energy Storage.

[69] Matos CR, Carneiro JF, Pereira da Silva P, Henriques CO (2021). A GIS-MCDA approach addressing economic-social-environmental concerns for selecting the most suitable compressed air energy storage reservoirs. Energies (Basel).

[70] Mrówczyńska M (2021). Scenarios as a tool supporting decisions in urban energy policy: The analysis using fuzzy logic, multi-criteria analysis and GIS tools. Renew. Sustain. Energy Rev..

[71] Ayodele TR, Ogunjuyigbe ASO, Odigie O, Munda JL (2018). A multi-criteria GIS based model for wind farm site selection using interval type-2 fuzzy analytic hierarchy process: The case study of Nigeria. Appl. Energy.

[72] Atici KB, Simsek AB, Ulucan A, Tosun MU (2015). A GIS-based multiple criteria decision analysis approach for wind power plant site selection. Util. Policy.

[73] Feizizadeh B, Jankowski P, Blaschke T (2014). A GIS based spatially-explicit sensitivity and uncertainty analysis approach for multi-criteria decision analysis. Comput. Geosci..

[74] Tarkowski R (2017). Perspectives of using the geological subsurface for hydrogen storage in Poland. Int. J. Hydrog. Energy.

[75] Czapowski G (2019). Perspektywy lokowania kawern magazynowych wodoru w pokładowych wystąpieniach soli kamiennych górnego permu (cechsztyn) w Polsce–ocena geologiczna. Biuletyn Państwowego Instytutu Geologicznego.

[76] Lankof L, Tarkowski R (2023). GIS-based analysis of rock salt deposits’ suitability for underground hydrogen storage. Int. J. Hydrog. Energy.

[77] Kaleem W, Tewari S, Fogat M, Martyushev DA (2023). A hybrid machine learning approach based study of production forecasting and factors influencing the multiphase flow through surface chokes. Petroleum.

[78] Motlagh ZK, Derakhshani R, Sayadi MH (2023). Groundwater vulnerability assessment in central Iran: Integration of GIS-based DRASTIC model and a machine learning approach. Groundw. Sustain. Dev..

[79] Azarafza M, Hajialilue Bonab M, Derakhshani R (2022). A deep learning method for the prediction of the index mechanical properties and strength parameters of marlstone. Materials.

[80] Tewari, S. & Dwivedi, U. D. A novel automatic detection and diagnosis module for quantitative lithofacies modeling. In *Society of Petroleum Engineers - Abu Dhabi International Petroleum Exhibition and Conference 2018, ADIPEC 2018* (2019). 10.2118/192747-ms.

[81] Beskopylny AN (2022). Concrete strength prediction using machine learning methods CatBoost, k-nearest neighbors, support vector regression. Appl. Sci. (Switzerland).

[82] Cemiloglu A (2023). Support vector machine (SVM) application for uniaxial compression strength (UCS) prediction: A case study for Maragheh Limestone. Appl. Sci. (Switzerland).

[83] Nakamura K (2023). A practical approach for discriminating tectonic settings of basaltic rocks using machine learning. Appl. Comput. Geosci..

[84] Nanehkaran YA (2023). Riverside landslide susceptibility overview: leveraging artificial neural networks and machine learning in accordance with the United Nations (UN) sustainable development goals. Water.

[85] Jalaee SA (2021). A novel hybrid method based on Cuckoo optimization algorithm and artificial neural network to forecast world’s carbon dioxide emission. MethodsX.

[86] Jalaee MS, Shakibaei A, Ghaseminejad A, Jalaee SA, Derakhshani R (2021). A novel computational intelligence approach for coal consumption forecasting in Iran. Sustainability (Switzerland).

[87] Dorogush, A. V., Ershov, V. & Gulin, A. CatBoost: gradient boosting with categorical features support. *arXiv preprint *arXiv:1810.11363 (2018).

[88] Lankof L, Tarkowski R (2023). GIS-based analysis of rock salt deposits’ suitability for underground hydrogen storage. Int. J. Hydrog. Energy.

[89] Zaresefat M (2022). Identification of suitable site-specific recharge areas using fuzzy analytic hierarchy process (FAHP) technique: a case study of Iranshahr Basin (Iran). Air Soil Water Res..

[90] Zaresefat M, Derakhshani R, Nikpeyman V, GhasemiNejad A, Raoof A (2023). Using artificial intelligence to identify suitable artificial groundwater recharge areas for the Iranshahr Basin. Water (Switzerland).

[91] Jalaee MS, Ghaseminejad A, Jalaee SA, Zarin NA, Derakhshani R (2022). A novel hybrid artificial intelligence approach to the future of global coal consumption using whale optimization algorithm and adaptive neuro-fuzzy inference system. Energies (Basel).

[92] Bahmani M, GhasemiNejad A, Robati FN, Zarin NA (2020). A novel approach to forecast global CO2 emission using Bat and Cuckoo optimization algorithms. MethodsX.

