# Supplementary material for: Artificial intelligence-driven assessment of salt caverns for underground hydrogen storage in Poland
Source: Sci Rep. 2024 Jun 20;14:14246. doi: 10.1038/s41598-024-64020-9 (PMC11190257; doi:10.1038/s41598-024-64020-9)
Supplement: Supplementary file 1 — Supplementary Information. [file 41598_2024_64020_MOESM1_ESM.docx]

**Supplementary**

**Implementation Details**

We have developed a site-selection methodology for hydrogen storage in salt caverns, leveraging spatial data and machine learning (ML). The research conducted by Lankof & Tarkowski^1^, employed a multi-criteria decision-making analysis based on the AHP to select the optimal location for hydrogen storage in salt caverns within the analyzed rock salt deposit. This type of analysis relies on the subjective assessment of experts who determine the weights of individual criteria (in this case, raster maps) during their pairwise comparisons.

Using machine learning (ML) to analyze the data, unlike the research conducted by LL and RT, would enable the selection of the best locations for hydrogen storage without the need for a multi-criteria decision analysis procedure, thereby eliminating the subjective element of decision-making. This methodology for identifying suitable locations for underground hydrogen storage involves several steps:

1. **Criteria for Choosing Suitable Areas:**
   - We considered two main groups of criteria:
     - **Exclusion Criteria**: These help us exclude certain areas from further analysis.
       - We excluded protected areas like natural reserves (including National forests, Special protection areas, Protected areas, Conservation areas, and Ecological sites).
     - **Evaluation Criteria**: These help us classify the remaining areas.
       - We looked at storage capacity, Water Reservoir, Accessibility, Natural gas pipeline, Geological exploration, Energy consumption, and Landuse.
2. **Creating Maps:**
   - We made twelve maps for each criterion (Exclusion and Evaluation Criteria) mentioned above, as illustraited in Figure 2.
     - Storage capacity is expressed as hydrogen energy that can be stored in the rock salt bed per area. Values correspond to the storage capacity after the first filling of the cavern.
     - Water reservoir map has been shown in a hydrological map of rivers and reservoirs at each location.
     - Accessibility map has been depicted by providing items like roads and railroads.
     - Natural gas pipeline map has been prepared to see the better accessibility to the available gas pipeline.
     - Geological exploration map shows the areas with the salt caverns that have been the target geological formation for reservation of the Hydrogen in this area and also includes the deep borehole locations.
     - Energy consumption map shows the energy consumption data in the study area.
     - Landuse map is provided to show the landscape elements such as residential buildings, restricted, recreational, industrial areas, cropland, forests, grassy areas, and barren and shrubland.
     - The other five exclusion criteria maps, including National forest, Special Protection area, Protected area, Conservation area, and Ecological site, show the protected areas around the rock salt deposits.
   - All these twelve maps were developed with an exact resolution of 100 points per 1 km^2^, meaning that a single pixel in the raster had dimensions of 100*100m. So, considering these 12 maps, we had 12 values for each point of the study area.
   - The next step of the methodology entails the transformation of basic maps (to quantify individual criteria) and the presentation of these in the form of raster maps with numerical values. These values were the starting point for calculations carried out using artificial intelligence, aimed at identifying the best locations for underground hydrogen storage facilities in salt caverns in the analyzed area. Maps showing roads, gas pipelines, water, and borehole locations required transformation. This was based on proximity maps, showing the distance between selected elements at each point on the map. However, due to their numerical approach, some maps like storage capacity, landuse and energy consumption did not require transformation. Raster maps are a set of pixels with assigned coordinates and values of the analyzed criteria.
3. **Normalization and Analysis:**
   - We normalized the values taken from raster maps to facilitate comparative analysis.
   - We did not apply any weight to these values.
   - We employed twelve standardized raster maps as input features for machine learning (ML) analysis.
   - This dataset was divided into a training set, constituting 70% of the total data, and a validation-testing set for the remaining 30%.
   - We employed the suitability map developed by Lankof & Tarkowski^1^ as the target for ML algorithm calibration.
   - The ML algorithm with the highest accuracy was selected for the final implementation.
   - The resultant suitability map for underground hydrogen storage within the rock salt layer was generated based on the data processed by the chosen ML algorithm.

**Reference**

1. Lankof, L. & Tarkowski, R. GIS-based analysis of rock salt deposits’ suitability for underground hydrogen storage. *Int J Hydrogen Energy* **48**, (2023).
